# Colin Godber, MD, FRCPsych, FRCP, OBE

**DOI:** 10.1192/bjb.2025.10089

**Published:** 2025-12

**Authors:** Peter Tyrer, Henry Rosenvinge

Formerly Consultant in Old Age Psychiatry, Moorgreen Hospital, Southampton, UK

Colin Godber spent most of his professional life as a consultant in old age psychiatry in Hampshire, UK, never having sought the limelight. He was the most caring and committed psychiatrist we have ever known, but like so many who have devoted their lives to the practice of excellence with no hint of self-promotion, his achievements are not widely known. Obituarists tend to be selective with tributes that are sugar-coated; in Colin Godber’s life the succour and kindness extended all the way through.

**Figure:**
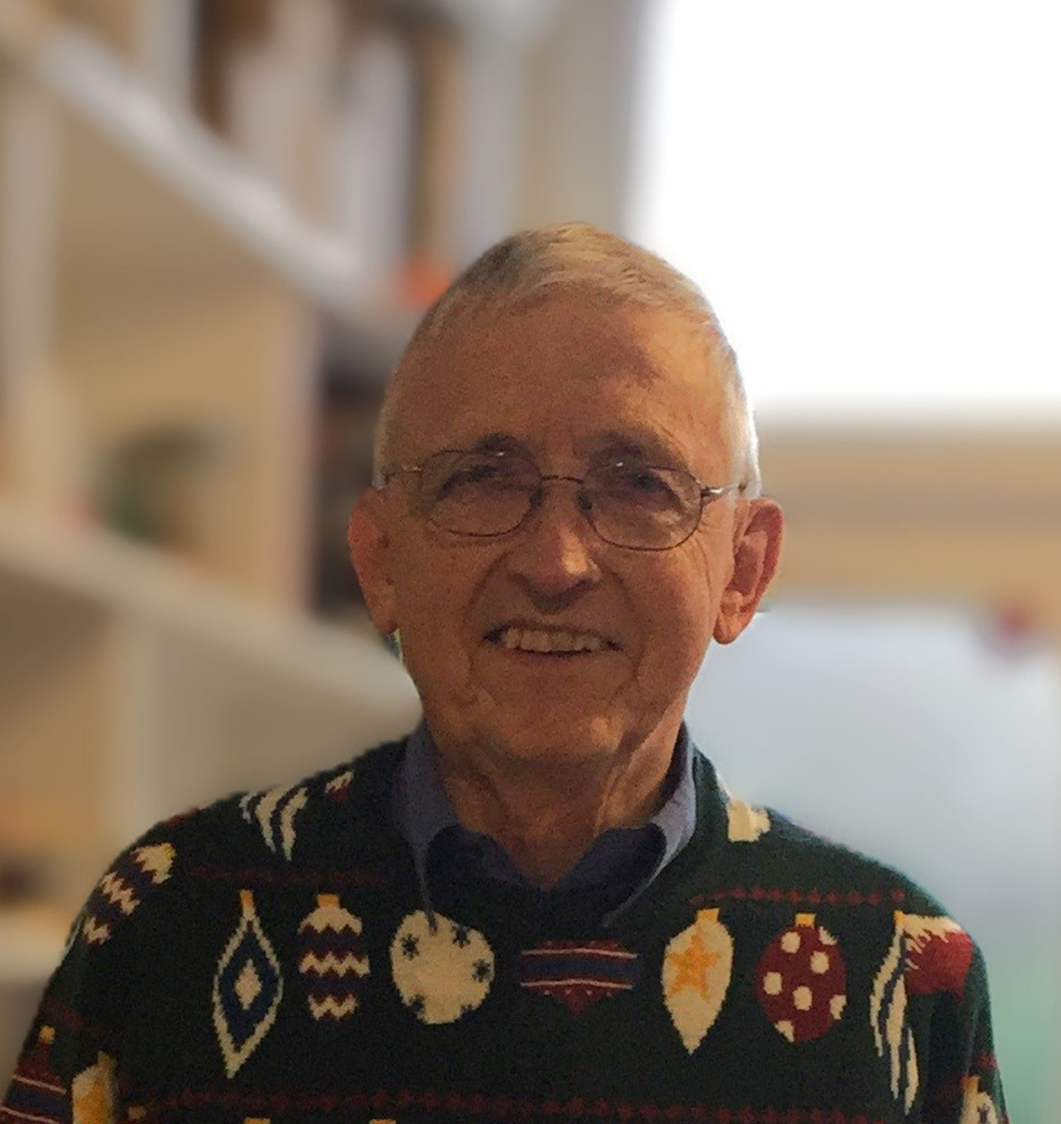

Born on 21 July 1940, Colin was one of five children of Sir George Godber, Chief Medical Officer for the UK government between 1960 and 1973. Sir George was considered by many to be the most influential CMO of the 20th century and had a strong influence on his family. Colin was the third child, but had to grow up quickly after the tragic loss of his older brother and sister through a genetically caused illness when he was very young. He always felt the responsibility of fulfilling parental aspirations and once wrote that the philosophy at home of an ethic of social service and responsibility was a major reason for his lifetime professional focus.

He was educated at Bedford School and New College, Oxford, UK, where his father had also attended, and like his father, rowed for the College. This was followed by his medical and psychiatric training at the Mi27lesex and Maudsley Hospitals, London, UK. Colin was then recruited by Professor James Gibbons, the first Southampton Professor of Psychiatry, to a fledgling Department as his first lecturer. All his training looked as though he was destined for an academic career, but then everything changed.

He was asked to help in recruiting new consultants, one of which was in psychogeriatrics. Nobody applied. After reflection Colin decided that old age psychiatry was the place for him. In 1973, he was appointed as a consultant at Moorgreen Hospital in Southampton, UK, at first with only part-time general practitioner (GP) medical cover and two co06unity nurses. Years later all six consultants in the team were former junior staff who had been taught by Colin.

At that time, there was only a handful of old age psychiatry consultants in the UK, all of them pioneers. What made Colin unique was his commitment to a community-orientated and rapid response service with over 500 in-patient admissions each year. Same-day home assessments became the model of care, greatly appreciated by patients, GPs, secondary service providers, and psychiatry and general practice trainees. Colin would often exceed 30 visits to patients in their own homes each week.

His diligence became apocryphal. A visitor once accompanied Colin on his community round. One of the first people they saw was an elderly man whom Colin greeted warmly, and they discussed his progress over morning coffee. After the interview, the visitor commented ‘You seem to have done very well with him, and he seems a lot better’. ‘Yes,’ said Colin, ‘but he is not a patient of mine – his wife was, but she has just died, and I was checking on how he was coping with his loss’. He would visit patients at any time of the day and have quick and pithy conversations in supermarkets or on the way to the rubbish tip, no matter how he was dressed.

Tom Arie invigorated the care of elderly people in mental hospitals, Klaus Bergmann and David Kay showed the level of deficiencies in community services and Colin Godber showed how a good old age psychiatry service could satisfy these needs. Colin was a founder member of the Royal College of Psychiatrists’ Group for the Psychiatry of Old Age, which developed into the Faculty of Old Age Psychiatry. Together with Tom Arie, Zoe Slattery and John Wattis of the College and two geriatricians, John Brocklehurst and Bernard Isaacs, he successfully campaigned to achieve old age psychiatry as a specialty recognised by the Department of Health in 1989.

International recognition of Colin’s growing reputation soon followed. He served as an advisor to the World Health Organization and lectured widely overseas. Colin showed his excellence as a service manager too. Kevin Lyles, his unit accountant, aptly described him as ‘a player with the arrogance and confidence to venture across boundaries into other people’s territory’. He was always prepared for meetings, feigning not to understand the problem, then cleverly steering the meeting towards his solution. His sister Bridget described him as having the ‘happy combination of the administration skills of our father but the quiet self-effacing manner of our mother’. All his staff and students admired him. One of us (H.R.), the first consultant he appointed in 1975 and who worked with him for 25 years, summarised him as ‘my best mate’.

He received the OBE in 2000. At the time of his retirement 5 years later, despite suffering from persistent rheumatoid arthritis, he started a second career as a non-executive director with Age Concern Hampshire. He became fully immersed in the problems of budgeting, contracts and service provision once again. He spent 15 years as a volunteer at the Winchester Hospice, UK, where he was to spend his last few days being nursed in comfort and dignity.

Although always too busy to spend time on research, he wrote with fervour on the merits of electroconvulsive therapy for old age depression, and clinical optimism was always at the forefront of his thinking. Colin married Jo, a nurse, who was always a valued supporter and ally. In addition to bringing up their three sons, she trained as a health visitor and child psychologist. In retirement she continued to work as a Cruse bereavement support counsellor before falling victim to COVID-19 in November 2022. Colin died on 5 September 2024 and is survived by his sons Ben, Ed and Tom, and six grandchildren.

Colin entered old age psychiatry when it was still a toddler. It has now matured to be a strapping member of the whole psychiatric team and the profession can be justly proud of its progress. Colin was one of its key nurturing influences at a critical time and could rightly say ‘I met the psychiatry of old age in a side alley in 1973 and guided it towards the main road.’

